# Convolutional Neural Network Models Help Effectively Estimate Legume Coverage in Grass-Legume Mixed Swards

**DOI:** 10.3389/fpls.2021.763479

**Published:** 2022-01-11

**Authors:** Ryo Fujiwara, Hiroyuki Nashida, Midori Fukushima, Naoya Suzuki, Hiroko Sato, Yasuharu Sanada, Yukio Akiyama

**Affiliations:** ^1^Hokkaido Agricultural Research Center, NARO, Sapporo, Japan; ^2^BANDAI NAMCO Research Inc., Tokyo, Japan

**Keywords:** convolutional neural network models, legumes, grass-legume mixed swards, image analysis, unmanned aerial vehicle

## Abstract

Evaluation of the legume proportion in grass-legume mixed swards is necessary for breeding and for cultivation research of forage. For objective and time-efficient estimation of legume proportion, convolutional neural network (CNN) models were trained by fine-tuning the GoogLeNet to estimate the coverage of timothy (TY), white clover (WC), and background (Bg) on the unmanned aerial vehicle-based images. The accuracies of the CNN models trained on different datasets were compared using the mean bias error and the mean average error. The models predicted the coverage with small errors when the plots in the training datasets were similar to the target plots in terms of coverage rate. The models that are trained on datasets of multiple plots had smaller errors than those trained on datasets of a single plot. The CNN models estimated the WC coverage more precisely than they did to the TY and the Bg coverages. The correlation coefficients (*r*) of the measured coverage for aerial images vs. estimated coverage were 0.92–0.96, whereas those of the scored coverage by a breeder vs. estimated coverage were 0.76–0.93. These results indicate that CNN models are helpful in effectively estimating the legume coverage.

## Introduction

Grass-legume mixtures are applied in a forage production to obtain a greater productivity and a higher nutritive value of forage. Compared with the grass monocultures, pasture yields improve in grass-legume mixed swards owing to nitrogen fixation by legumes (Lüscher et al., 2014; Suter et al., 2015). In mixed swards, nitrogen fixed by forage legumes from the atmosphere is transferred to non-legumes (Pirhofer-Walzl et al., 2012; Thilakarathna et al., 2016). Furthermore, nitrogen fixed by legumes in mixed swards is higher than that in the legume monocultures (Nyfeler et al., 2011). Consequently, grass-legume mixtures improve the productivity of swards. Feeding the forage legumes to livestock can enhance the milk yields and the nutritional quality (Dewhurst et al., 2009; Peyraud et al., 2009). Therefore, the forage obtained from the grass-legume mixed swards can also be beneficial in terms of feed quality. In Japan, timothy (*Phleum pratense* L., TY) and white clover (*Trifolium repens* L., WC) are widely utilized for grass-legume mixed swards.

Legume proportion in mixed swards fluctuates dynamically over time, and patterns of the fluctuation vary depending on the proportion of seeds in the mixture, soil fertility, and climate conditions (Rasmussen et al., 2012; Suter et al., 2015; Bork et al., 2017). To maintain an appropriate legume proportion, it is crucial to obtain suitable forage varieties and to ensure proper management of grass-legume mixtures. Therefore, in breeding and in cultivation research, the evaluation of legume proportions is necessary. In Japan, for several times a year, the forage breeders score the coverage of grass and legume as an indicator of legume proportion. However, estimating the legume proportion in swards through observations of researchers may be subjective, and separating the legumes from the non-legumes by harvest measurements is time-consuming.

Unmanned aerial vehicles (UAVs) make it possible to obtain big data from images in a short time and conduct precise image analysis. The use of UAVs is becoming widespread in various fields, including agricultural analysis (Colomina and Molina, 2014). Analysis of UAV-based aerial images is also applied to remote sensing of sward height and of biomass in grasslands (Michez et al., 2019).

The image analysis method for objective and time-efficient estimation of legume proportions has been examined. Himstedt et al. (2012) applied color segmentation with legume-specific thresholds in hue saturation and light (HSL) color space to images of swards and predicted legume coverage and dry matter contribution. McRoberts et al. (2016) extracted local binary patterns (LBP), one of the texture descriptors in image classification, and developed regression models to estimate grass composition in alfalfa-grass fields. Mortensen et al. (2017) distinguished plant material from soil with excess green (ExG) and excess red (ExR) vegetation indices calculated from the RGB images, and detected the legume leaves with an edge detection and a reconstruction using flood filling.

In addition to image analysis methods using local color indices or feature extractors, convolutional neural networks (CNNs) are utilized in image classification or object detection. Convolutional neural network (CNNs) are a multi-layer neural networks equipped with convolutional and pooling layers, and they have a strong ability of complicated feature recognition (LeCun et al., 2015). There have been many studies on the application of CNNs in various aspects of agriculture (Kamilaris and Prenafeta-Boldú, 2018), including crop grain yield estimation (Yang et al., 2019), weed detection in grasslands (Yu et al., 2019a,b), and crop pest recognition (Thenmozhi and Srinivasulu Reddy, 2019; Li et al., 2020).

Some studies have applied CNNs to the estimation of legume proportion, especially in methods involving semantic segmentation. Semantic segmentation is a pixel-to-pixel classification task. The fully convolutional network (FCN) has been developed for solving the problem of segmentation (Shelhamer et al., 2017). Skovsen et al. (2017) trained an FCN architecture to distinguish clover, grass, and weed pixels. Larsen et al. (2018) examined the data collection workflow with UAVs and demonstrated the network (Larsen et al., 2018). Bateman et al. (2020) developed a new network for semantic segmentation, called the local context network, which distinguished clover, ryegrass, and the background more accurately than the FCN. Despite these studies, few examples of CNN application in the estimation of a legume proportion are available, and the knowledge required to develop the CNN models has not been fully accumulated. Besides, understanding how to develop models suitable to various fields and comparison between the models using different datasets may be useful.

GoogLeNet is a CNN model equipped with Inception modules and is the winner of the ImageNet Large Scale Visual Recognition Challenge (ILSVRC) 2014 competition (Szegedy et al., 2015). Mehdipour Ghazi et al. (2017) demonstrated a plant identification with three CNN architectures, GoogLeNet, AlexNet, and VGGNet, using the dataset of LifeCLEF 2015. In the study, VGGNet was the most accurate, AlexNet was the fastest in terms of training, but GoogLeNet achieved competitive results both in terms of accuracy and of training speed. Because GoogLeNet has a well-balanced architecture, we considered it desirable to develop and compare multiple models.

In the current study, the CNN model estimating the coverage area of timothy, white clover, and the background (Bg) from UAV-based aerial images was trained by fine-tuning GoogLeNet. Multiple CNN models were trained on different datasets under the same conditions, and their accuracies were compared. To evaluate the usability of the CNN models, the correlations between the scored coverage by a breeder, measured coverage using aerial images, and estimated coverage by the CNN models were analyzed.

## Materials and Methods

### Field Experiment and Data Collection

The field experiment and data collection were conducted at Hokkaido Agricultural Research Center (Hokkaido, Japan). Each of three white clover cultivars under a variety test (“cultivar A,” “cultivar B,” and “cultivar C”) was mix-sowed with timothy on May 31, 2016. The plot size was 2 m × 3 m for each replicate (four replicates with three cultivars), and the amount of seeds sown was TY: 150 g/a and WC: 30 g/a in each plot. The plot design was determined using a randomized block design.

Coverage estimation, through scoring by a breeder and image acquisition with a UAV, was conducted 2 years after the seeding. A coverage score (%) for the three categories (TY, WC, and Bg) was assigned by a breeder on October 9, 2018 (scored coverage). The UAV-based aerial image of each plot was taken using DJI Phantom 4 Pro (SZ DJI Technology Co., Ltd., Shenzhen, China) on October 10, 2018, 14 days after the 3rd cutting of that year. The camera of Phantom 4 Pro had lens with an 8.8 mm focal length and a 1″ CMOS 20 M sensor. The UAV hovered above each plot at an altitude of 4 m and took one image. The image was stored as a Digital Negative (DNG), a format of RAW images. The ground sample distance was ∼1 mm/pixel. The images were imported to a personal computer and were adjusted with Photoshop CC (Adobe, San Jose, CA, United States). After auto-correction, the images were converted to PNG format. The images were cropped to the region of the plots and were keystone-corrected with the perspective crop tool. The size of the cropped images was approximately 2,000 × 3,000 pixel.

**FIGURE 1:**
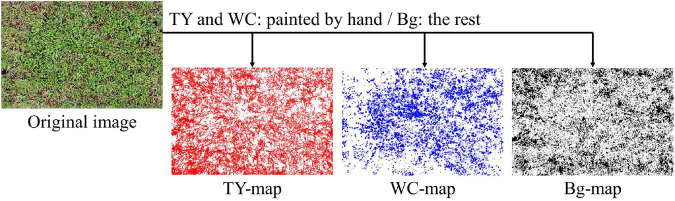
Example of timothy (TY), white clover (WC), and background (Bg) maps generated from a UAV-based aerial images.

On each image of plots, blank layers for three categories (TY, WC, and Bg) were generated. Pixels belonging to TY and WC were painted on its respective layer with Photoshop CC using a pen display (Wacom Cintiq 16, Saitama, Japan) by hand. Pixels not belonging to TY or WC were painted as Bg category. Therefore, each layer acted as a map for that category (Figure 1). The layers were output as PNG files. The rates (%) of painted pixels on the maps were calculated with Python 3.6.8 (Python Software Foundation, 2018), Numpy 1.19.4 (Harris et al., 2020), and Pillow 8.0.1 (Clark, 2021). Thus, the percentage of the painted pixels represents the coverage rate of each category measured on the aerial image (measured coverage).

### Training and Evaluation of the Convolutional Neural Network Models

**FIGURE 2:**
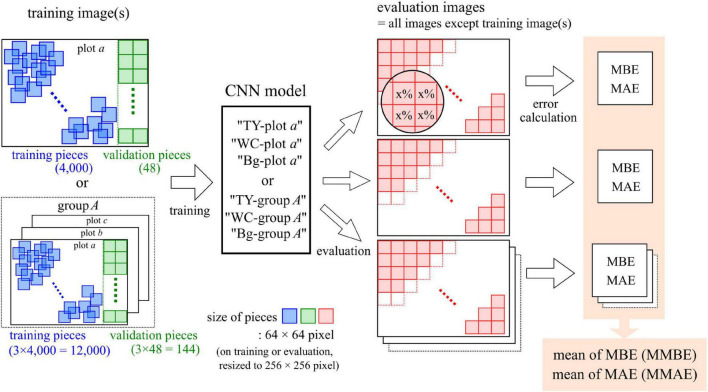
The process of training and evaluation of the convolutional neural network (CNN) models.

The process of training and evaluation of the CNN models is shown in Figure 2. This process was conducted on a Windows 10 PC using a Core i9 7900X CPU, an RTX 2080 Ti GPU, and 64 GB RAM. The environment for CNN was constructed with Anaconda (Anaconda Software Distribution, 2021) using Python 3.6.2 (Python Software Foundation, 2017), CUDA 10.1 (NVIDIA Corporation, Santa Clara, CA, United States), cuDNN 7.5 (NVIDIA Corporation), Chainer 6.5.0 (Tokui et al., 2019), and cupy 6.5.0 (Okuta et al., 2017). Our previous research (Akiyama et al., 2020) was referenced in training the CNN models.

### Formation of Training Datasets and Training of the Convolutional Neural Network Models

As the training dataset for a model, image pieces were cut from an aerial image of one plot or from aerial images of three plots in a group. For training, 4,000 pieces of 64 × 64-pixel images were randomly cut from the region, excluding 128 pixels on the right side of the plot image. For validation, 48 pieces of 64 × 64-pixel size images were cut from 128 pixels on the right side, in order, from the upper left without overlaps. On the maps of TY, WC, and Bg, the rate of painted pixels (*r*_*p*_) was calculated at the location of each piece. The *r*_*p*_ of each category was divided into 21 classes set every 5%, as shown in Table 1 (the handling of values on the boundary is due to the behavior of the round function of Python). Sixty-four by sixty-four pixel-sized pieces were resized to 256 × 256 pixels by the nearest neighbor interpolation. These pieces and classes of TY, WC, and Bg coverage were used as the training dataset for a CNN model.

**TABLE 1:** Classification of the rate of positive pixel in each region.

Class	Class value (%)	Class	Class value (%)	Class	Class value (%)
0≤*r*_*p*_≤0.025	0	0.325 < *r*_*p*_ < 0.375	35	0.675≤*r*_*p*_≤0.725	70
0.025 < *r*_*p*_ < 0.075	5	0.375≤*r*_*p*_≤0.425	40	0.725 < *r*_*p*_ < 0.775	75
0.075≤*r*_*p*_≤0.125	10	0.425 < *r*_*p*_ < 0.475	45	0.775≤*r*_*p*_≤0.825	80
0.125 < *r*_*p*_ < 0.175	15	0.475≤*r*_*p*_≤0.525	50	0.825 < *r*_*p*_ < 0.875	85
0.175≤*r*_*p*_≤0.225	20	0.525 < *r*_*p*_ < 0.575	55	0.875≤*r*_*p*_≤0.925	90
0.225 < *r*_*p*_ < 0.275	25	0.575≤*r*_*p*_≤0.625	60	0.925 < *r*_*p*_ < 0.975	95
0.275≤*r*_*p*_≤0.325	30	0.625 < *r*_*p*_ < 0.675	65	0.975≤*r*_*p*_≤1	100

GoogLeNet, with the weights pre-trained on ImageNet, was trained on these datasets. The hyper-parameters were learning rate: 0.01, batch size: 32, optimizer: momentum Stochastic Gradient Descent (SGD; momentum = 0.9), and training epochs: 500. The accuracy (the rate of correct prediction on 21-classes classification) of each training model was checked with validation datasets every 1,000 iterations. The weight was saved during the validation. After training was completed, the weight with the highest accuracy upon validation was selected as the model for that dataset. The training mentioned above was conducted using datasets of 16 image sets ( = 12 plots + 4 groups) across three categories (TY, WC, and Bg). A model trained on a dataset of a plot was named “(TY, WC, or Bg)-plot *a*” (*a* = plot code), and a model of a group was named “(TY, WC, or Bg)-group *A*” (*A* = group number). The properties of the models are shown in Table 2.

**TABLE 2:** The properties of the models trained in this study.

Model’s name (xx = TY, WC, or Bg)	Plot(s) for training	Number of pieces	
		Training	Validation
xx-plot 1-1	plot 1-1	4,000	48
xx-plot 1-2	plot 1-2	4,000	48
xx-plot 1-3	plot 1-3	4,000	48
xx-plot 1-4	plot 1-4	4,000	48
xx-plot 2-1	plot 2-1	4,000	48
xx-plot 2-2	plot 2-2	4,000	48
xx-plot 2-3	plot 2-3	4,000	48
xx-plot 2-4	plot 2-4	4,000	48
xx-plot 3-1	plot 3-1	4,000	48
xx-plot 3-2	plot 3-2	4,000	48
xx-plot 3-3	plot 3-3	4,000	48
xx-plot 3-4	plot 3-4	4,000	48
xx-group 1	plot 1-1, 2-1, 3-1	12,000	144
xx-group 2	plot 1-2, 2-2, 3-2	12,000	144
xx-group 3	plot 1-3, 2-3, 3-3	12,000	144
xx-group 4	plot 1-4, 2-4, 3-4	12,000	144

### Evaluation of the Convolutional Neural Network Models

The trained model was evaluated using the evaluation images, which were the images not used in the training of each model. Images 64 × 64 pixels in size were cut from the evaluation images without overlaps (the remainder at the end of the image was not used) and resized to 256 × 256 pixels. One thousand two hundred to one thousand five hundred pieces of image were cut from each image. These pieces were applied to the CNN model to obtain the predicted class value of each piece. On the maps of TY, WC, and Bg, the class value on the location of each piece was measured in the same way as on the training datasets. Using the predicted class value and the measured class value, the mean bias error (MBE) and the mean absolute error (MAE) were calculated (Willmott, 1982; Willmott and Matsuura, 2005) as follows:


(1) $$\begin{array}{c} MBE=\frac{1}{n}\sum_{j=1}^{n}\left(P_{j}-O_{j}\right) \end{array}$$

(2) $$\begin{array}{c} MAE=\frac{1}{n}\sum_{j=1}^{n}\left|P_{j}-O_{j}\right| \end{array}$$

where *n* is the number of cases in the evaluation (pieces cut from an image), *P*_*j*_ is the predicted class value, and *O*_*j*_ is the observed class value.

The MBE indicates the bias of the model. Particularly, when the MBE is positive, the model tends to over-estimate; and when it is negative, the model tends to under-estimate. The MAE indicates the magnitude of the prediction error of the model.

One set of MBE and MAE values was obtained when one model was employed to predict pieces that were cut from an image of one plot (one-model-to-one-plot prediction). For the evaluation of the models, the means of MBEs (MMBE) and MAEs (MMAE) were calculated for each model using the following formulae:


(3) $$\begin{array}{c} MMBE=\frac{1}{N}\sum_{i=1}^{N}MBE_{i} \end{array}$$

(4) $$\begin{array}{c} MMAE=\frac{1}{N}\sum_{i=1}^{N}MAE_{i} \end{array}$$

where *N* is the number of images used for evaluating the model (all images except the ones used in the training), and *MBE*_*i*_ and *MAE*_*i*_ are the MBE and MAE of each one-model-to-one-plot prediction, respectively.

### Estimation of the Measured and Scored Coverage

**FIGURE 3:**
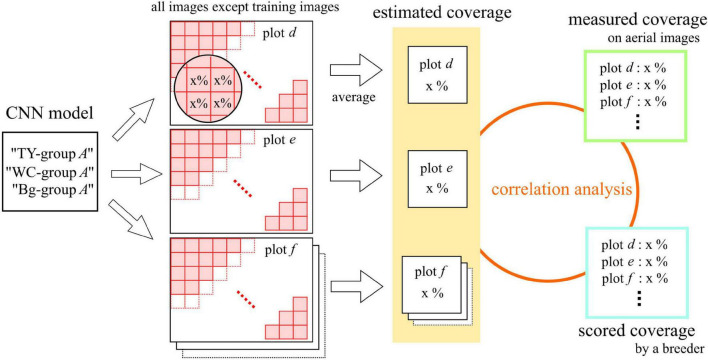
The estimation process of measured and scored coverage by the CNN models.

The estimation process is shown in Figure 3. In each one-model-to-one-plot prediction, the predicted values of the pieces were averaged. The average was regarded as the estimated coverage of the plot by the model. The model estimated the coverage of the plots from the dataset of a group, except of the ones used for the training. For the verification of the CNN models, the correlations between scored coverage, measured coverage, and estimated coverage by the model were analyzed.

In previous studies, the background has been distinguished from plant bodies using the excess green (ExG) and excess red (ExR) vegetation indices (Meyer and Neto, 2008; Mortensen et al., 2017). In our datasets of the 12 plots, the rate of pixels with zero or negative excess green minus, that of pixels with zero or negative excess red indices (ExG – ExR), was calculated as the estimated coverage of the background, as per the method of Meyer and Neto (2008). For comparison with the CNN method, the correlation of the measured coverage on aerial images vs. the estimated coverage with ExG – ExR was analyzed.

### Evaluation of the Convolutional Neural Network Models for Predicting Legume Coverage Using Different Datasets by Grass or Legume Species

Datasets that are different to those used in training by grass or by legume species mix-sowed in the field were used to evaluate the accuracy of legume coverage prediction by the trained CNN models. On the date which the image was taken, the UAV used in aerial photographing, and the pasture species of grass, orchard grass (OG), and legume, WC or red clover (RC), are shown in Table 3. These images were taken over the fields in Hokkaido Agricultural Research Center (mentioned above). As shown in the table, DJI Phantom 4 RTK (SZ DJI Technology Co., Ltd., Shenzhen, China) was used for both OG-RC 3 and OG-RC 4, while Phantom 4 Pro was used for the others. The spec of the camera of Phantom 4 RTK is the same as that of the Phantom 4 Pro. The OG-RC 3 and OG-RC 4 were taken from the same plot on different dates, while the plots of other images were different to each other. The legume coverage maps of these images were generated (as shown in Figure 1). Images 64 × 64 pixels in size were cut from the generated images and predicted by the CNN model trained for each group. The coverage of RC was also predicted with the WC models. In the same way, MBEs and MAEs were calculated for the evaluation of the models.

**TABLE 3:** Status of the images used in evaluation of legume prediction on fields differing in grass or legume species.

Image	Grass	Legume	Date taken	Lapsed days after cutting	UAV
OG-WC 1	OG	WC	2018/5/31	16 days	DJI Phantom 4 Pro
OG-WC 2	OG	WC	2018/5/31	16 days	DJI Phantom 4 Pro
OG-RC 1	OG	RC	2019/7/19	53 days	DJI Phantom 4 Pro
OG-RC 2	OG	RC	2019/7/19	53 days	DJI Phantom 4 Pro
OG-RC 3	OG	RC	2020/10/19	20 days	DJI Phantom 4 RTK
OG-RC 4	OG	RC	2020/10/26	27 days	DJI Phantom 4 RTK

## Results

### Scored and Measured Coverage on Each Plot

The scored coverage by the breeder and the measured coverage on aerial images (measured using painted maps) are shown in Table 4. The sum of the measured coverage of the three categories (TY, WC, and Bg) on each plot was not precisely 100% because the maps of the categories were painted individually. The scored coverage tended to be higher in WC and lower in Bg, compared with the measured coverage. In every category, the range of the scored coverage was wider; that is, the breeder scored plots without much difference in the dynamically measured coverage. The correlation coefficient of the scored and measured coverages was high in WC but not in TY and Bg.

**TABLE 4:** The scored coverage by a breeder and the measured coverage using aerial images.

			Scored coverage by a breeder (%)			Measured coverage using painted maps (%)		
Plot	Group	Cultivar	TY	WC	Bg	TY	WC	Bg
1-1	1	Cultivar B	50	50	0	41.4	27.8	31.5
1-2	2	Cultivar A	70	25	5	47.4	13.6	38.9
1-3	3	Cultivar C	45	35	20	38.3	16.8	44.8
1-4	4	Cultivar C	45	40	15	49.2	13.8	37.2
2-1	1	Cultivar C	70	25	5	37.7	13.2	46.5
2-2	2	Cultivar B	40	45	15	40.3	17.2	42.5
2-3	3	Cultivar B	45	50	5	37.6	26.8	35.6
2-4	4	Cultivar A	50	50	0	37.5	18.2	44.3
3-1	1	Cultivar A	70	30	0	42.2	22.0	35.8
3-2	2	Cultivar C	50	40	10	46.3	17.3	36.4
3-3	3	Cultivar A	55	45	0	41.2	20.9	37.8
3-4	4	Cultivar B	25	70	5	37.0	31.6	31.4
		Mean	51.3	42.1	6.7	41.3	19.9	38.6
		Range	45.0	45.0	20.0	12.2	18.3	15.1
Correlation coefficient (*r*) of scored vs. measured coverage						0.29	0.79	0.35

### Evaluation and Comparison of the Convolutional Neural Network Models

**FIGURE 4:**
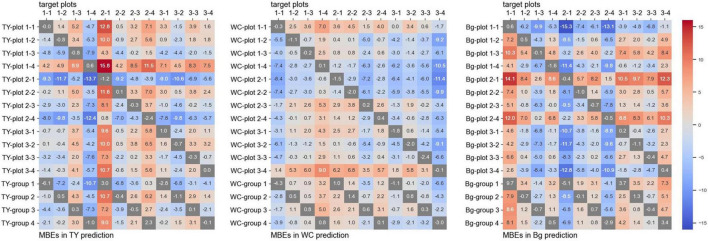
The mean bias errors (MBEs) for every one-model-to-one-plot prediction.

**FIGURE 5:**
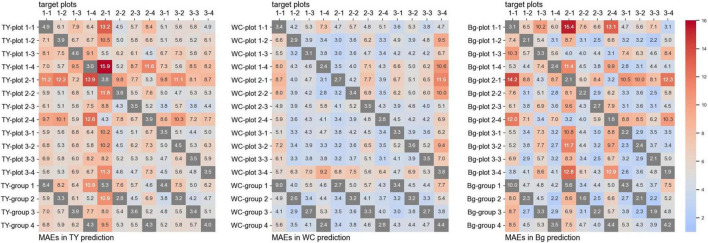
The mean average errors (MAEs) for every one-model-to-one-plot prediction.

The training time for the CNN models from one plot was approximately 4,000 s, and that from a group (three plots) was approximately 12,000 s. The MBEs for every one-model-to-one-plot prediction are shown in Figure 4, and the MAEs are shown in Figure 5. In these figures, the MBEs and the MAEs for predicting the images used in training each model are also shown in gray squares. The models trained on data from the plots, whose measured coverage rates were high (such as “TY-plot 1-4,” “WC-plot 3-4,” and “Bg-plot 2-1”; Table 4), tended to over-estimate; they had positive and high MBEs for predicting other plots. Contrary to this, the models trained on data from plots with low coverage rates (“TY-plot 2-1,” “WC plot 2-1,” and “Bg-plot 1-1”) tended to under-estimate. The prediction errors (MAE) were high when these over or under-estimating models were used.

For prediction using the models trained on plots, whose measured coverage rates were close to the target (e.g., model: “TY-plot 2-1” and target: plot 2-4, and vice versa), the MBEs were close to zero, and the MAEs were low. The MAEs for predicting WC coverage of plot 3-4, the plot with high WC coverage, were high in many models but were lower in the model trained on data from another high-coverage plot (such as “WC-plot 1-1” and “WC-plot 2-3”). The Plot 2-2, which shared “cultivar B” but did not have high WC coverage, was predicted with high MAEs by “WC-plot 1-1,” “WC-plot 2-3,” and “WC-plot 3-4.” Therefore, in this case, the main factor influencing the tendency of the model to predict with high MAEs was the WC coverage, not the cultivar.

**FIGURE 6:**
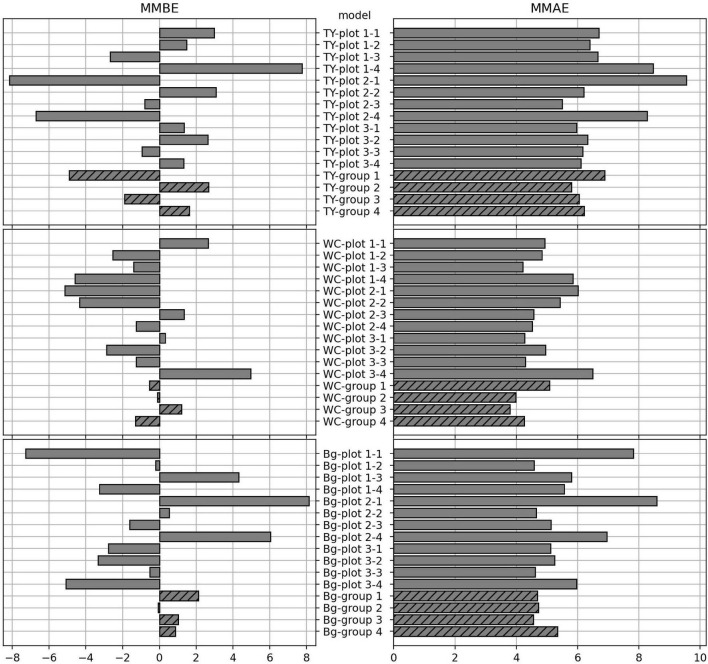
The mean of mean bias errors (MMBE) and the mean of mean average errors (MMAE) for predicting the coverage of the plots by the CNN models. The calculation for each model doesn’t include the MAEs and the MBEs on predicting the images used in the training.

The MMBE and the MMAE are shown in Figure 6. The calculation of MMBE and MMAE of each model did not include the MAEs and the MBEs for predicting the images used in the training. Therefore, the MMBE and the MMAE are the averages of each row without the gray squares in Figures 4, 5. Overall, the MMAE was lower in WC than that in TY and Bg. Compared with the models trained on data from a plot, the MMAE of the models trained on data from a group was lower. Moreover, though the MMAEs of some models trained on data from a plot were extremely high, the MMAEs of the models trained on data from a group were relatively stable. This showed that the models trained on datasets representing multiple conditions could predict wider target images accurately. When the MMAE of a model was high, such as in the case of “TY-plot 2-1,” “WC-plot 3-4,” and “Bg-plot 2-1,” the absolute value of the MMBE was also high, that is, such a model tended to over or under-estimate.

### Estimation of the Measured and Scored Coverage

**FIGURE 7:**
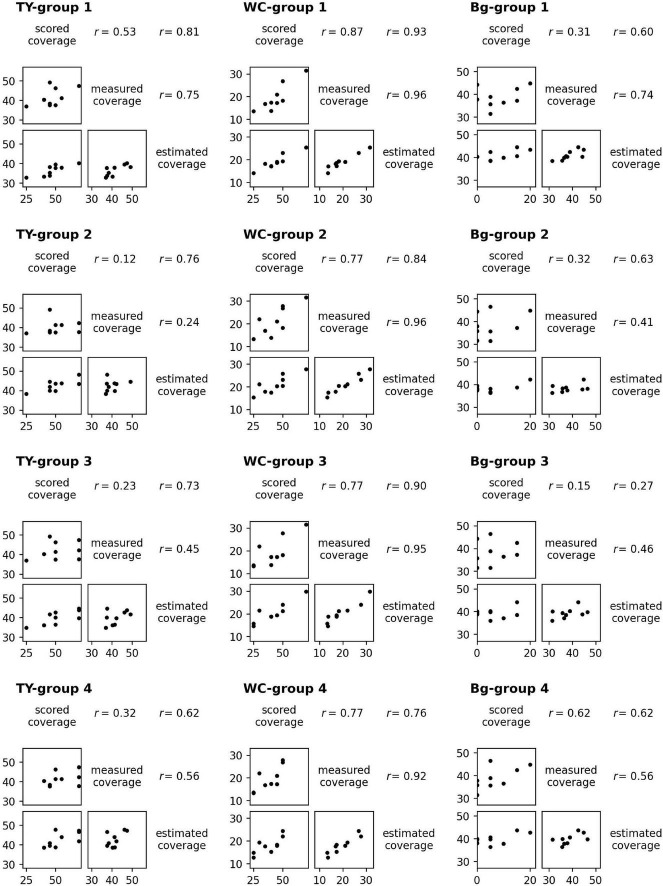
The scatter plots and the correlation coefficients (r) of scored coverage by a breeder, measured coverage on aerial images, and estimated coverage by CNN models trained from groups.

For the models of TY, WC, and Bg trained on a dataset from each group, the scatter plots and the correlation coefficients (*r*) of scored coverage, measured coverage, and estimated coverage are shown in Figure 7. The results were different between models even in the scored vs. measured coverage pair because the plot data used to train the models were omitted in each pair. For WC, the correlation coefficients in every pair of scored, measured, and estimated coverage were high: *r* = 0.92–0.96 in measured vs. estimated coverage (the highest was “WC-group 2”: *r* = 0.961), and *r* = 0.76–0.93 in scored vs. estimated coverage (the highest was “WC-group 1”: *r* = 0.934). For TY and Bg, the correlation coefficients of measured vs. estimated coverage were lower, *r* = 0.24–0.75, in TY and *r* = 0.41–0.74 in Bg. In TY, the correlation coefficient of scored vs. estimated coverage exceeded that of measured vs. estimated coverage with every model.

**FIGURE 8:**
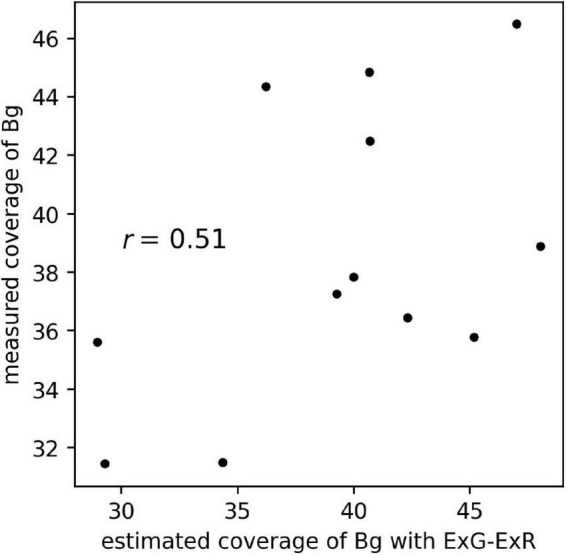
The scatter plot of estimated coverage of Bg with ExG – ExR and measured coverage of Bg on aerial images of 12 plots.

The scatter plot of the estimated coverage of Bg with ExG – ExR and the measured coverage of Bg on aerial images of the 12 plots is shown in Figure 8. The correlation coefficient of the estimated coverage with ExG – ExR vs. measured coverage was 0.51, the same extent as with the CNN Bg- models (*r* = 0.41–0.74).

### Evaluation of Legume Coverage Prediction Using Different Datasets by Grass or Legume Species

**FIGURE 9:**
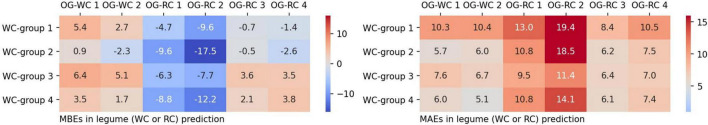
The MBEs and the MAEs for predicting legume coverage of different images by grass or legume species to those used in training.

**FIGURE 10:**
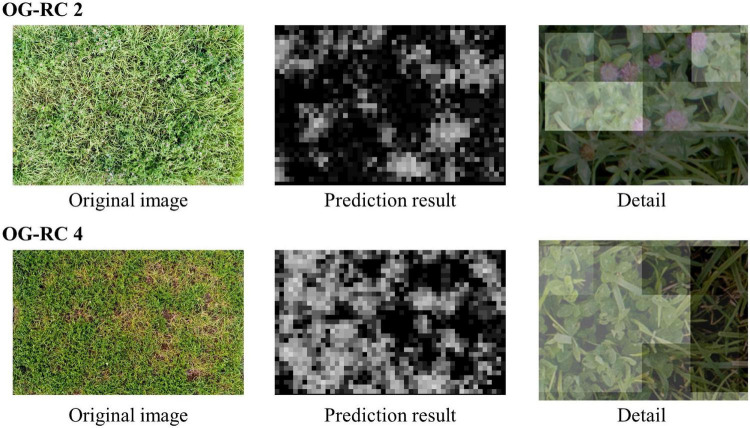
The original plot images and the prediction results of OG-RC 2 and OG-RC 4. “Prediction result” is the result map of the legume (RC) coverage prediction by “WC-group 3” illustrated in grayscale (when a sheet is close to white, the predicted class value is high). “Detail” is an enlarged view of the original image on which the prediction result map overlapped (the opacity of the result map is adjusted in overlapping).

Using the WC model trained on the dataset of each group, legume coverage on images of the OG-WC and OG-RC fields was predicted. The MBEs and MAEs for the prediction are shown in Figure 9. The coverage of the WC of OG-WC 1 and 2, taken on fields with a different grass species (OG), was predicted with MAEs lower than 10 by “WC-group 2,” “WC-group 3,” and “WC-group 4,” though the MAEs increased by several points from those shown in Figure 5. When coverage of a different legume species (RC) was predicted by the WC-models, RC coverage of OG-RC 1 and 2 was predicted with relatively high MAEs and negative MBEs, that is, the models tended to under-estimate. In contrast, the RC coverage of OG-RC 3 and 4 was predicted with lower MAEs. Both the OG-RC 3 and OG-RC 4 differed from OG-RC 1 and 2 in season and year of the images being taken (Table 3). The difference between OG-RC 2 and OG-RC 4 in original images, prediction results by “WC-group 3,” and details of the prediction are shown in Figure 10.

## Discussion

The generalization of the CNN model is a major problem. The “WC-group 3” model used in this study was trained on the images of plot 2-3, a plot with high WC coverage, and other plots with a low coverage (Table 4). Consequently, the MAEs for predicting both high and low-coverage plots were suppressed (Figure 5), and the MMAE of the model was low (Figure 6). This model is likely to succeed in generalization. It is suggested that a wide distribution of coverage rate in training datasets leads to high accuracy of predicting different types of plots. However, “WC-group 1,” trained on datasets including that from a high-coverage plot, plot 1-1, predicted other high-coverage plots with high MAEs. The reason of this may be the deficiency in fitting the model to the training datasets because this model also predicted plot 1-1, used in training the model, with a high MAE. A wide distribution of the coverage in training datasets, and a thorough training to fit the model to the datasets could be needed.

**FIGURE 11:**
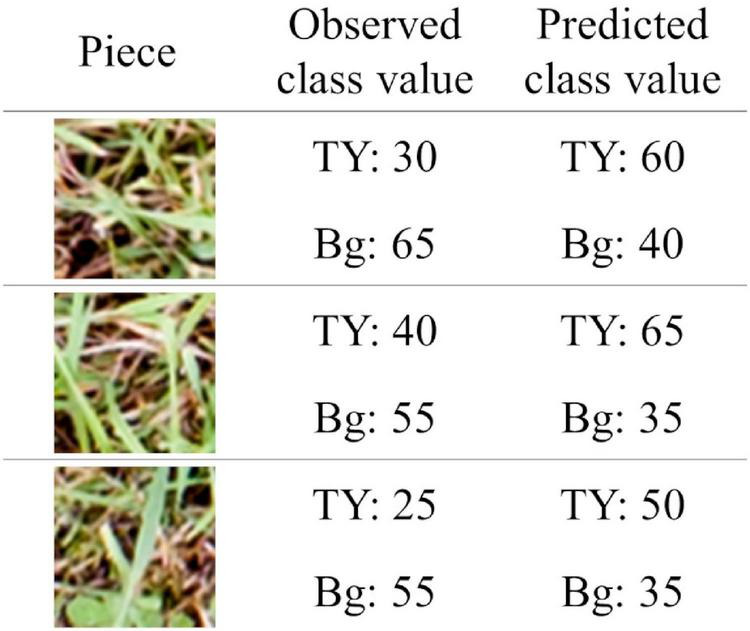
The examples of the piece-level prediction in which TY and Bg were confused. The pieces were cut from plot 2-1. The coverage of TY was predicted by “TY-group 2” and that of Bg was predicted by “Bg-group 2.”

Judging by the correlation data shown in Figure 7, coverage estimation of legume by CNN models is likely to be easier than that of the grass or background. The reason for this may be the difference in the shape of leaves. Particularly, legume leaves are wider than those of grasses, thus, CNN can fully extract the features of legume leaves from the aerial images. Moreover, in this study, there were cases where distinguishing TY from the background was difficult on paintings of the location because there were withered TY leaves on the mixed swards in autumn. In such cases, the training datasets had some uncertainty. This may be one of the reasons why the coverage estimation of TY was inaccurate. The “TY-group 2” over-estimated the TY coverage of plot 2-1 (MBE: 10.7, MAE: 10.9), while the “Bg-group 2” under-estimated Bg coverage of plot 2-1 (MBE: −8.5, MAE: 8.6), as shown in Figures 4, 5. The examples of the piece-level prediction are shown in Figure 11. In these examples, including withered TY leaves on sheets, TY class values were over-estimated and Bg class values were under-estimated. When maps of each category for training were painted on hand, the withered TY leaves were not painted as TY, and thus, painted as Bg. These withered TY (painted as Bg) areas are likely to be predicted as TY due to the shapes of the leaves. In this way, TY and Bg could be confused by the CNN models.

The background, the location with no plants present, lacks a characteristic shape. Feature extraction of the background by the CNN models may be difficult because the background does not have a unique shape. Using our datasets, the prediction of background coverage with ExG – ExR (Meyer and Neto, 2008) was not accurate (Figure 8). For the estimation of background coverage, other methods that involve vegetation indices or machine learning may be needed.

The comparison of the multiple models shown in Figures 4, 5 can be a variation of the cross validation with MBE and MAE, though the validation in our case was different to common cross validation in that the size of our validation datasets was larger than that of the training datasets. On the models generalized sufficiently, the prediction errors of validation datasets are near the prediction errors of training datasets in cross validation. From this point of view, the WC prediction models in our study were well-generalized, compared with those of TY and of Bg.

The scored coverage by a breeder reflects the 3D features that the aerial 2D images cannot grasp. Therefore, the scored coverage is not necessarily inferior to the measured coverage on images, though the scored coverage is subjective. It is likely that the CNN models can estimate both measured coverage and scored coverage for legumes based on the high correlations of predicting WC coverage observed (Figure 7). On the other hand, in TY and Bg, the correlations of scored vs. measured coverage were low. This may be due to the difference between the appearance of TY or Bg to a breeder and that from a UAV. It seems to be difficult to produce an estimation of a breeder by predicting TY or Bg coverage from images using CNN models.

However, in TY models, the correlations of scored vs. estimated coverage were higher than those of measured vs. estimated coverage (Figure 7). This means that the CNN models estimated the scored coverage more precisely even though the models were trained on measured coverage data. In general, the CNNs were likely to be trained on characteristic parts of images and made predictions using such parts, as demonstrated through visual explanation methods such as the Grad-CAM (Selvaraju et al., 2020). For the prediction of TY coverage, the CNN models may be trained mainly on data of the characteristic parts ( = typical parts for TY) and may predict a high coverage using the plot images of such parts. Breeders also look at characteristic parts in plots and score the coverage. This may be the reason why the correlations of the scored coverage by a breeder vs. the estimated coverage by the CNN models were higher. These results suggest that the CNN models make predictions using the data generated through human decision-making more precisely than using data measured mechanically. Additional research is needed to confirm this.

When the WC coverage from the OG-WC images was predicted by the CNN models trained with TY-WC images, an increase in MAEs was limited for “WC-group 2,” “WC-group 3,” and “WC-group 4” (Figure 9). It appears that the WC-models trained with TY-WC images are applicable to WC coverage prediction of mixed swards with a different grass species. On the other hand, when the RC coverage from OG-RC images was predicted by the WC-models, the MAEs increased on OG-RC 1 and 2 (Figure 9). In these images, there were pieces with RC presence that were predicted to have low legume coverage, as shown in “Detail” of OG-RC 2 in Figure 10. In OG-RC 1 and 2, the RC leaves stood upwardly, and thus, looked sharper. Such RC leaves had different shapes on imaging to WC leaves. In contrast, in OG-RC 3 and 4, RC leaves looked similar to WC leaves. This may be the reason why the WC-models predicted the RC coverage of OG-RC 1 and 2 with higher MAEs, and that of OG-RC 3 and 4 with lower MAEs. For training the model to predict RC coverage accurately, training datasets, which cover leaf shapes of various RC conditions, should be needed.

In this study, for comparing multiple models using different training datasets in the same conditions, adjustment of the architectures and hyperparameters of the CNN was not conducted. Adequate accuracy for coverage estimation of WC was achieved in this condition. The following points can be considered for further improvement of the models: (1) The architecture of the CNN: Yu et al. (2019a) reported that AlexNet and VGGNet achieved higher precision values for weed detection in perennial ryegrass than GoogLeNet. The CNN models for the coverage estimation of mixed swards can be improved with architectures other than GoogLeNet. (2) The optimizer used for training the CNN model: Momentum SGD was used as the optimizer in our study, but other optimizers, such as AdaGrad (Duchi et al., 2011) and Adam (Kingma and Ba, 2014), can be used. Adjustment of hyperparameters, including optimizers, may improve the coverage estimation models of grass-legume mixed swards. (3) The problem setting: In the predictions in this study, a 21-class classification was applied to the CNN models because GoogLeNet has been developed to address the issue of classification. The CNN models for regression problems, however, are also buildable. There are precedents for this in crop yield prediction (Nevavuori et al., 2019) in and maize tassels counting (Lu et al., 2017). The development of CNN regression models that predict coverage as a continuous value may be promising.

In previous studies, methods involving semantic segmentation have mainly been applied to the prediction of a legume proportion using CNNs (Skovsen et al., 2017; Larsen et al., 2018; Bateman et al., 2020). On the other hand, in this study, class values of coverage in separate regions were predicted. Using this method, many pieces of images for training can be obtained from a fixed number of aerial images. Moreover, prediction errors may be suppressed because values of coverage are predicted directly, and not by interposing the classification on each pixel. So far, the superiorities of these methods are not clear. Additionally, although the measured coverage on aerial images and the scored coverage by a breeder were used as indicators of legume proportion in this study, yield-based indicators such as dry matter yield are also likely to be useful. Comparative studies between the prediction methods of legume proportion are required.

The CNN system to investigate a small experimental field was developed in this study because of the difficulty to take high resolution images for a large field. However, the investigation system for the large production field is important. The capability to capture a large field mainly depends on the performance of UAVs; examples are flight time, the camera sensor size, and the camera lens. As the technology of UAVs becomes more advanced, this CNN system may be useful for the large production field in the future.

Multiple CNN models estimating the coverage of timothy (TY), white clover (WC), and the background (Bg) from UAV-based aerial images were trained and were compared. The accuracy of the CNN models used in our study was affected by the coverage on the plots in the training datasets, and thus, it was suggested that a wide distribution of the coverage rate in the training datasets was important for the generalization of the model. The WC coverage, both the measured coverage on aerial images and the scored coverage by a breeder, was precisely estimated by the CNN models.

The CNN model trained on data from a group of the three plots was shown to be useful for the estimation of the WC coverage. It is expected that further works based on the methods in this study will generate a practical system to estimate the coverage in grass-legume mixed swards.

## Data Availability Statement

The raw data supporting the conclusions of this article will be made available by the authors, without undue reservation.

## Author Contributions

RF analyzed the results and wrote the manuscript. HN, MF, and NS conceived the idea and proposed the method. HS and YS performed the experiments. YA designed the experiments and analyzed the results. All authors contributed to the article and approved the submitted version.

## Conflict of Interest

HN, MF, and NS were employed by company BANDAI NAMCO Research Inc. The remaining authors declare that the research was conducted in the absence of any commercial or financial relationships that could be construed as a potential conflict of interest.

## Publisher’s Note

All claims expressed in this article are solely those of the authors and do not necessarily represent those of their affiliated organizations, or those of the publisher, the editors and the reviewers. Any product that may be evaluated in this article, or claim that may be made by its manufacturer, is not guaranteed or endorsed by the publisher.

## Abbreviations

Bg: background
CNN: convolutional neural network
ExG: excess green
ExR: excess red
FCN: fully convolutional network
HSL: hue, saturation, and lightness
MAE: mean absolute error
MBE: mean bias error
MMAE: mean of MAE
MMBE: mean of MBE
OG: orchard grass
RC: red clover
TY: timothy
UAV: unmanned aerial vehicle
WC: white clover.
